# Characterization of a Low-Cost Plastic Fiber Array Detector for Proton Beam Dosimetry

**DOI:** 10.3390/s20205727

**Published:** 2020-10-09

**Authors:** Cigdem Ozkan Loch, Michael Alexander Eichenberger, Michele Togno, Simon Pascal Zinsli, Martina Egloff, Angela Papa, Rasmus Ischebeck, Antony John Lomax, Peter Peier, Sairos Safai

**Affiliations:** 1 Department of Large Scale Research Facilities, Paul Scherrer Institut, 5232 Villigen, Switzerland; eicmicha@student.ethz.ch (M.A.E.); szinsli@student.ethz.ch (S.P.Z.); rasmus.ischebeck@psi.ch (R.I.); 2 Center for Proton Therapy, Paul Scherrer Institut, 5232 Villigen, Switzerland; michele.togno@psi.ch (M.T.); martina.egloff@psi.ch (M.E.); tony.lomax@psi.ch (A.J.L.); sairos.safai@psi.ch (S.S.); 3 Department for Research with Neutrons and Muons, Paul Scherrer Institut, 5232 Villigen, Switzerland; angela.papa@psi.ch; 4 Laboratory Ionising Radiation, Federal Institute of Metrology (METAS), 3003 Bern-Wabern, Switzerland; peter.peier@metas.ch

**Keywords:** scintillator, fiber, protons, electrons, beam monitoring, dosimeter

## Abstract

The Pencil Beam Scanning (PBS) technique in proton therapy uses fast magnets to scan the tumor volume rapidly. Changing the proton energy allows changing to layers in the third dimension, hence scanning the same volume several times. The PBS approach permits adapting the speed and/or current to modulate the delivered dose. We built a simple prototype that measures the dose distribution in a single step. The active detection material consists of a single layer of scintillating fibers (i.e., 1D) with an active length of 100 mm, a width of 18.25 mm, and an insignificant space (20 μm) between them. A commercial CMOS-based camera detects the scintillation light. Short exposure times allow running the camera at high frame rates, thus, monitoring the beam motion. A simple image processing method extracts the dose information from each fiber of the array. The prototype would allow scaling the concept to multiple layers read out by the same camera, such that the costs do not scale with the dimensions of the fiber array. Presented here are the characteristics of the prototype, studied under two modalities: spatial resolution, linearity, and energy dependence, characterized at the Center for Proton Therapy (Paul Scherrer Institute); the dose rate response, measured at an electron accelerator (Swiss Federal Institute of Metrology).

## 1. Introduction

In Pencil Beam Scanning (PBS) [1] dose is delivered to the patient by the magnetic scanning of successive energy layers of narrow, mono-energetic pencil beams, across the tumor volume. The flexibility provided by this technique, especially when combined with a treatment gantry that can rotate fully around the patient such that multiple treatment fields can be delivered from different directions [2], has led to this approach now being the most common proton therapy delivery modality, after the pioneering work of the Center of Proton Therapy (CPT) at the Paul Scherrer Institute [3]. Thanks to the flexibility in dose painting, proton therapy, and specifically PBS, has shown advantages, such as a decreased integral dose and better normal tissue sparing [4,5], for certain indications compared to the classical approach with photons [6].

The energy transferred to tissue by protons is inversely proportional to their velocity, thus the dose deposition varies as a function of the track length up to a maximum, which is called the Bragg peak. In PBS, a homogeneous dose to the target can be achieved by the superimposition of multiple Bragg peaks associated with different initial energy. In real clinical cases, proton energy and local fluences are necessarily highly modulated [2], and thus the energy spectrum of protons depositing dose at any given point can be complex and broad. In short, information on clinically relevant irradiation fields, with their widely varying proton energy spectra and differing contributions from secondary particles, needs to be well-mapped on a point-to-point basis in the delivered distribution. We intend to address this problem with the development of a stackable scintillator fiber-based dosimeter for measuring PBS proton fields as delivered in a clinical environment of the CPT. The scintillator fibers are made from doped polystyrene.

In the field of proton beam dosimetry, extensive research has been carried out in characterizing and evaluating plastic scintillators. Beddar et al. [7,8] demonstrated that characteristics such as water equivalence, fast response time, and dose-rate responses of plastic scintillators qualify them for external beam radiation therapy. At the CPT, the response of several detectors has been tested and characterized to understand their suitability for proton dosimetry [9,10,11]. Different types of scintillating fibers generally have different light emission spectra, light outputs, and levels of quenching. Safai et al. [9] have shown that we can take advantage of such differences to improve overall detector performance.

Research on singular plastic fibers and multiple ones has been covered by [12,13]. Plastic scintillating fibers have been used for a while now and stacked to give 2D [12,14,15] information on dose distribution, proving the performance of the fibers in beam monitoring for conventional radiotherapy and proton therapy, and also the feasibility of the stacking for 3D information. The fibers in these designs are 1 mm in size (square or circular), spaced by the same distance as their size. In some cases, the scintillators are coupled to clear plastic optical fibers and in other cases, read out directly. Reference [12] used a camera to readout the scintillators while [15] used photomultiplier tubes (PMTs) to detect the signal from each fiber. In-depth study for the application of plastic fibers to in vivo dosimetry in high dose rate brachytherapy was carried out by [16]. Aside from fibers, large plastic scintillators for quality assurance purposes were also proposed [17].

Several detector types used in proton dosimetry for therapeutic applications, such as plastic and inorganic scintillators, gel detectors and Gafchromic films, have a response that shows some level of dependence on the proton energy [18]. For example, LET response variability of Gafchromic EBT3 film from a Co-60 calibration in clinical proton beam qualities is demonstrated in [19,20]. The effect is particularly visible when such detectors are employed to measure the dose-depth curves of quasi-mono-energetic proton beams, which then exhibit a less pronounced peak dose compared to ionization chamber measurements. For plastic scintillators irradiated with protons, with variable linear energy transfer (LET), the light output is suppressed [21]. As protons slow down due to energy loss, energy transfer to the medium (with higher LET) increases, and a larger fraction of the energy is lost to interactions that do not emit light [17]. This leads to suppression in the relative light output in the Bragg peak [17]. This process is known as quenching [22], and is described by Birks’ law [23].

A low-cost prototype based on scintillating fibers was built into a 1D array, where one end of this array is bundled together into a random circular pattern. The detection medium is an imaging camera. The idea is to be able to orthogonally stack the fiber-array so as to simultaneously obtain information on the LET in the third dimension, depth.

This work aims to characterize the fiber array’s response to changes in energy and linearity with proton irradiation. The proton dose-depth profile measured with the prototype was compared to that measured with an ionization chamber, by comparing extracting the Bragg peak position. To do this, we translated images from the bundle end of the array to its line end and tested the feasibility of the reconstruction, which is explained in this paper. Additionally, the paper presents the calibration of such a detector at the Federal Institute of Metrology, Switzerland, which has a pulsed electron facility for dosimeter calibration purposes.

## 2. Materials and Methods

### 2.1. Setup at the CPT

The fiber array detector was based on scintillating plastic fibers and a CMOS based area scan camera (model: Basler ace acA1920-50gm). The camera featured a resolution of 2.3 MP and has a dark noise of 6.7 electrons, a quantum efficiency of 70%, and can be programmed for integration times from a few microseconds up to seconds, with a dynamic range of 8 to 12 bit [24]. Full frame images can be acquired at up to 50 frames per second in 8 bit, or 25 frames per second in 12 bit. A camera-based readout was preferred as enlarging the fiber array would not bring additional cost to the readout, as it most certainly would with a readout based on photomultiplier tubes.

**Figure 1:**
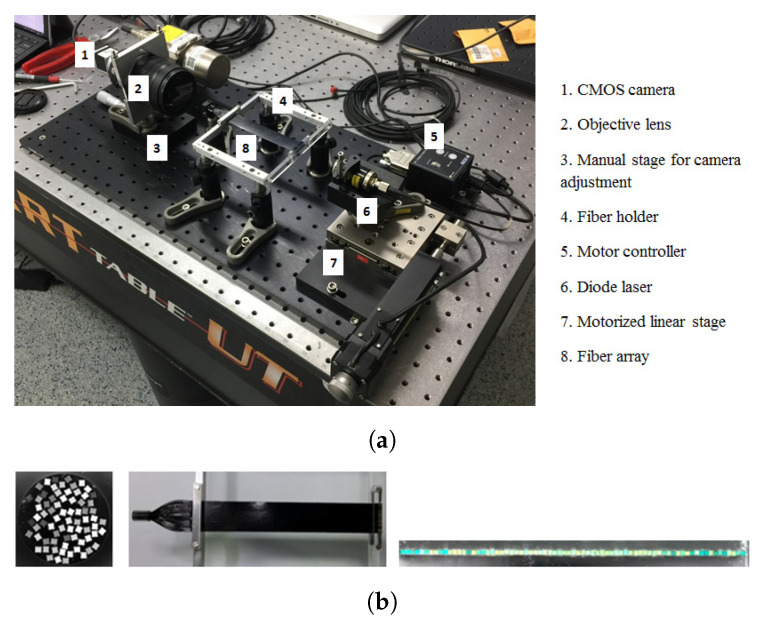
(**a**) Setup of the fiber array with the CMOS camera. (**b**) Picture of the fiber array on its holder with a picture of the bundled end of the array on the left and its flat end on the right.

The fiber array was made of a single layer of square fibers (BCF-20 from Saint Gobain Crystals, 250 × 250 μm${}^{2}$, 160 mm long [25]). Each fiber was coated with acrylic water-based paint (measured thickness around 10 μm), to avoid light leak between adjacent fibers. The fibers were glued together with optical cement [26] that also helped overcome the minor imperfections in the fibers to achieve a flatness of the order of 10 μm. The total width of the array is 18.25 mm. A picture of the setup can be found in Figure 1.

The end of the fiber array not exposed to the beam was bundled together in a circular pattern (Figure 1a) and imaged onto a CMOS imager. The fibers have a random arrangement at the bundle end (Figure 1b). Bundling was so as to avoid additional effort in pulling each fiber, one-by-one, and fastening into a holder especially machined to host each fiber. Bundling also allows us to easily image a large bundle or more than a single bundle onto the camera’s field of view. Images acquired with the CMOS camera are reconstructed quickly, as detailed later in this section, allowing the translation of a 2D image into a 1D profile.

**Figure 2:**
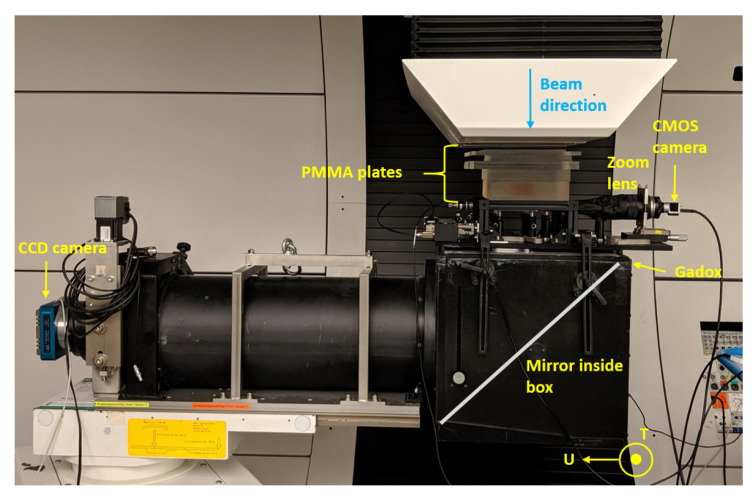
Picture of the lateral view of the setup at the Gantry. The CCD camera is located 1 m from the mirror at 45${}^{\circ }$ that reflects the light from the Gadox screen. The fiber array sits in the U direction, as indicated. The T direction is normal to the page.

For the measurement at Gantry 2 (CPT), the setup in Figure 1a was mounted on top of the in-house built imaging device available at Gantry 2. This device consists of a Gadolinium Oxysulfide (Gadox) scintillating screen, a 45${}^{\circ }$ mirror and a cooled CCD to read out the scintillating light. On top of the Gadox screen, there is a 4.5 mm thick PVC plastic cover used for protection and avoiding ambient light leaking into the camera. The purpose of using the CCD was mainly for cross-checking beam measurements. A lateral view of this setup is shown in Figure 2.

### 2.2. Setup at METAS

Next, the detector was taken to the Swiss Federal Institute of Metrology (METAS) for calibration. The electron accelerator of type Microtron generated a pulsed electron beam. The beam energy ranged 4–22 MeV and the typical pulse length was 3 μs. The electron beam was converted into reference electron or photon radiation fields of beam quality for dosimetry with a scattering target and a Bremsstrahlung target, respectively. Depending on the beam quality and the pulse repetition frequency, the dose rate can be tuned from a few mGy/min up to 60 Gy/min. The dose was applied at repetition rates from 1–250 Hz, which allowed us to study the dose rate response of the detector. The reference field size at 1000 mm distance from the scattering target was adjustable (0.5 × 0.5 cm${}^{2}$ to 20 × 20 cm${}^{2}$). The homogeneity of a 15 × 15 cm${}^{2}$ field in the centered 2 × 2 cm${}^{2}$ was at the percent level. A larger part of the beam is cut away with collimators, so small changes in the electron beam from the machine do not have a strong influence on the used beam at the experiment. For our measurements, the camera was externally triggered to allow the acquisition of an image every beam pulse delivered.

We chose to replace the Basler acA1920-50gm with the pco.edge 5.5 based on the scientific CMOS (sCMOS) sensor [27]. This camera was capable of frame rates up to 1 kHz at small regions of interest. The sCMOS sensor also had a low noise performance of 1.1 electrons and a dynamic range of 16 bit, which allowed us to study the single-pulse low-dose-rate response of the detector.

**Figure 3:**
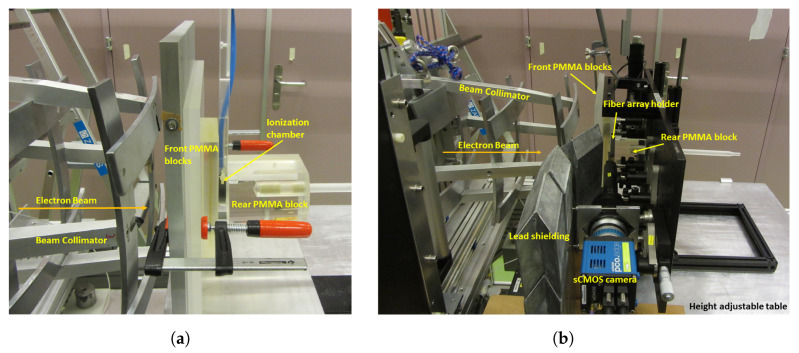
(**a**) Calibration setup for the ionization chamber in the water-equivalent setup in the air with 35 mm PMMA (Polymethyl methacrylate) slabs. (**b**) Picture of the fiber array detector setup mounted vertically at METAS.

Directly in front of the experimental setup is an Integrating Current Transformer (ICT) for measuring the electron beam current (0.1–2 mA) of every pulse. Before mounting the setup, the readings from the ICT were calibrated to give a dose reading per pulse. To calibrate the ICT reading from V to Gy, a calibrated ionization chamber (IC) was placed in the reference position in a water phantom and measurements were done under reference conditions. Data were then acquired at different dose rate values. Since the fiber array setup could not be immersed into water, water-equivalent perspex slabs were mounted in front of and behind the detector to mimic the water immersion scenario. Perspex slabs of 35 mm total thickness were mounted in front of and a large perspex slab was mounted behind the IC, as shown in Figure 3a. Comparing the calibration factors found in the two IC scenarios—immersion in water and sandwiched between perspex slabs—the calibration factors measured for 2.1 mGy/pulse were within 1.06% of each other. For a dose rate of 6.1 mGy/pulse, the calibration factors are within 0.3%. This means that sandwiching the fiber array detector between the same perspex slabs was sufficient to mimic the water immersion scenario.

For the measurements, the setup in Figure 2 was mounted vertically and just like the IC in Figure 3a, the same perspex slabs were then mounted around the detector. A lateral view of the final measurement setup at METAS is shown in Figure 3b.

### 2.3. Image Reconstruction

The bundling of the fibers at one end would eventually allow for imaging a multilayered array-detector with a single camera. Since the fibers have a random arrangement at one end, a reconstruction of the one-dimensional profile of the fiber array from a two-dimensional image of the bundle end is necessary.

If a single fiber of the array is irradiated, a distinct image of that fiber can be seen on the camera. If multiple fibers are irradiated simultaneously, the resultant image on the camera can be fully described as a linear combination of the images of individually irradiated fibers:

(1) A·x=b,

where, for a bundle of 72 fibers, *A* is a matrix of size 576,000 × 72, a subset of the 2.3 MP camera, which has the flattened image of each individual fiber (900 × 640 image to 576,000 × 1 vector) as columns, *b* is the resultant image seen on the camera as a flattened 576,000 vector (pixel values), and *x* is a vector containing the irradiation coefficient of each fiber. To determine *x*, we use least square approximation of the linear coefficients of the linear combination of all images. For example, if the *i*-th fiber is illuminated, this model returns the calibration image of the *i*-th fiber:

(2) $$\left(\begin{array}{ccc} Image_{1,1} & \cdots & Image_{1,72}\\ \vdots & \ddots & \vdots \\ Image_{576000,1} & \cdots & Image_{576000,72} \end{array}\right)\cdot \left(\begin{array}{c} 0\\ \vdots \\ 1\\ \vdots \\ 0 \end{array}\right)=\left(\begin{array}{c} Image_{i,1}\\ \vdots \\ Image_{i,576000} \end{array}\right)$$

In order to reconstruct the signal distribution of the fibers for a given bundle image, the linear equation A·x=b must be solved for *x*. In our setup, we used the Moore–Penrose inverse $A^{+}$ of *A*, to retrieve the least-square approximation $\hat{x}$ of *x*. Thus, for an arbitrary image captured by the camera, the corresponding signal distribution of the fibers is given by:

(3) $$\hat{x}=A^{+}\cdot b.$$

The advantage of this approach is that the pseudo-inverse $A^{+}$ can be computed in advance, allowing for fast runtime image reconstruction.

### 2.4. Position Calibration

**Figure 4:**
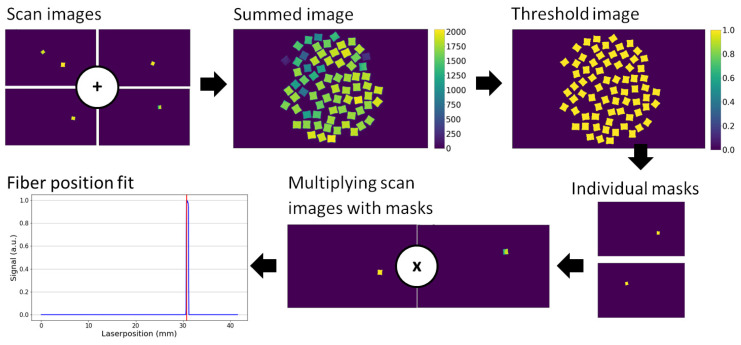
Images of the single fibers are summed to form the fiber bundle image, which is then used to make a binary mask of the bundle. From this binary mask, the single fiber masks are extracted. The single fiber masks are then multiplied with the scanned images and a 1D profile of the fiber location is found. The plot shows the found fiber position with respect to the UV diode laser position.

Images of every single fiber, illuminated one at a time, are required for calibration. For this reason, a software routine was written for the automatic alignment of the UV diode laser used to illuminate each fiber. This was done by scanning the entire fiber array with the laser and creating a summed image (Figure 4). Then, for each individual fiber, a binary mask was extracted. By extracting the illumination on the masked area for each fiber, a one-dimensional data-set of the fiber signal with respect to the laser position could be obtained. The ideal laser alignment for the calibration was found by peak fitting (Figure 4).

## 3. Results

### 3.1. Results from Measurements at CPT

For our measurement purposes, a 150 MeV proton beam spot was scanned with a step size of 2.5 mm to obtain two different beam fields: T-field is a 1D line of 41 spots that move across the width of the fiber array, and U-field is a 1D line of 41 spots that move along the length of the fiber array with a spacing of 2.5 mm and a single spot size of 2.83 mm rms, as shown in Figure 2. T and U directions are orthogonal to each other. In discrete spot scanning mode at Gantry 2, the beam current was suppressed, while the steering magnets moved from one set-point (spot position) to the next, resulting in ∼3 ms dead time between spots. The beam current at the exit of the treatment nozzle was set to ∼600 pA, which is well-comparable to the current used for clinical treatments.

**Figure 5:**
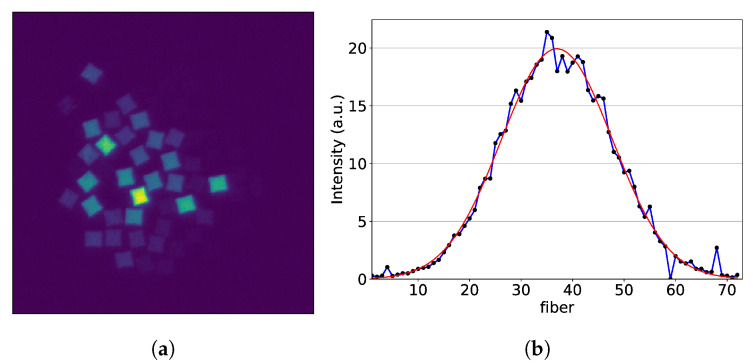
(**a**) Image of the fiber bundled captured by the CMOS camera. (**b**) 1D profile reconstructed from this image is shown on the right. Background has not been subtracted from this image.

With the beam in a U-field configuration, the image captured by the CMOS camera is shown in Figure 5a. Using the calibration matrix to reconstruct the 1D profile of the beam (Figure 5b), it can be seen that the beam has a transverse Gaussian shape.

A Gaussian fit to the data resulted in a standard deviation of 2.69 ± 0.13 mm. This agrees well with the nominal beam spot size of 2.83 mm.

**Figure 6:**
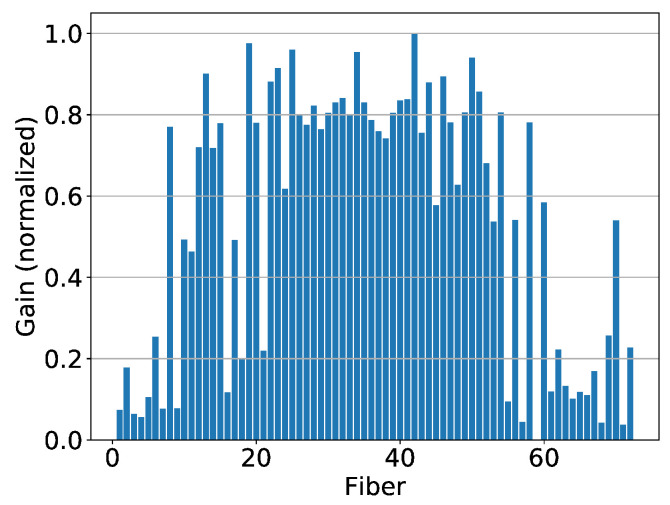
The plot shows the relative signal of one fiber to another, normalized to the intensity of the most intense fiber.

Homogenous illumination of the fibers showed the response variation between them, as shown in the plot in Figure 6. The intensity of each fiber was normalized to the most intense fiber to show a relative variation. In image reconstruction, this variation is normalized by the calibration matrix, as discussed in the section Materials and Methods.

**Figure 7:**
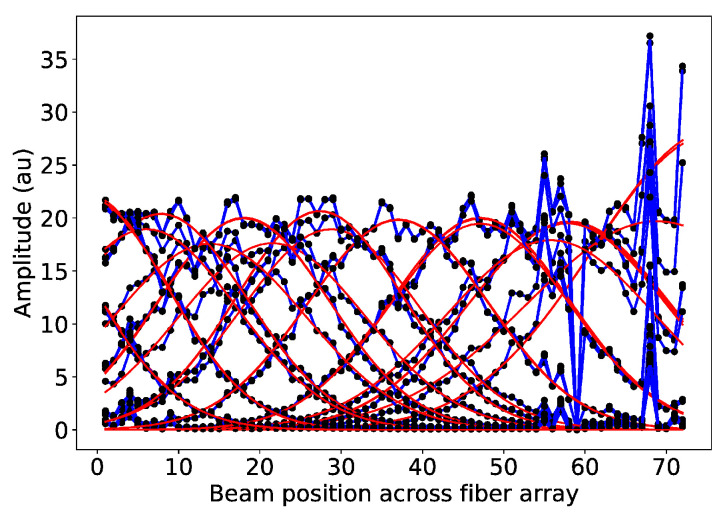
Beam motion capture during T-field irradiation. Background has been subtracted from the images. Camera exposure time was 100 ms whereas the beam delivery was 530 ms per each spot with an irradiation of 2 × $10^{9}$ protons/spot. The background was subtracted from each image before reconstruction to a 1D profile.

In a T-field configuration, we could capture the motion of the pencil beam moving across the width of the fiber array with a camera exposure of 100 ms. The 1D profiles reconstructed from each 2D image show the beam motion and its step size. Each profile was fit with a Gaussian and the average step size was found to be 2.55 mm ± 0.09 mm. Since the delivery of each spot, with 2 × $10^{9}$ protons per spot, takes about 530 ms, at the acquisition rate of 10 Hz the full spot profile is reconstructed at each expected position in at least four subsequent images. In the image sequence (Figure 7), some spots appear to be reconstructed at intermediate locations between the expected positions. This effect can be explained considering the interplay between the beam motion and the camera acquisition rate. Spots reconstructed at intermediate locations are the results of the convolution of two subsequent spots integrated over a single acquisition frame.

**Figure 8:**
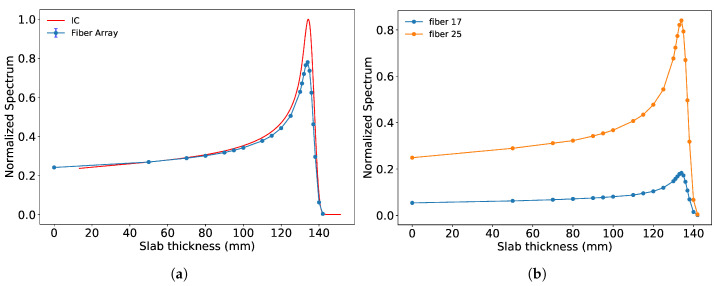
(**a**) Plot shows Bragg profiles reconstructed compared to the normalized ionization chamber (red). The fiber array profile was normalized to the ion chamber (IC) profile at 50 mm depth. (**b**) The plot shows Bragg profiles of individual fibers can also be extracted. Fibers 18 and 26 are shown, and their intensities have been normalized to the maximum intensity from a fiber in the array.

Dose-depth measurements were performed by shifting the energy along the Bragg curve using PMMA (Polymethyl methacrylate) plates of various thicknesses and piling them on top of each other above the detector. The T-field configuration of the beam was used as it features a uniform dose distribution along the width of the fiber array. Images of the beam were acquired with the fiber array setup for an acquisition time of 10 s. The Bragg curve was reconstructed by summing the entire image of the bundle and plotting with increasing slab thickness. The profiles acquired by the fiber array detector and ionization chamber (from a different measurement campaign) are shown in Figure 8a. The two curves exhibit a difference of the Bragg peak by 0.3 mm, when extracting the 80% distal dose fall-off. The Bragg information is available for each fiber and can be extracted using the calibration matrix, as shown for two fibers in Figure 8b. No quench factor correction was implemented for the Bragg profile reconstruction.

**Figure 9:**
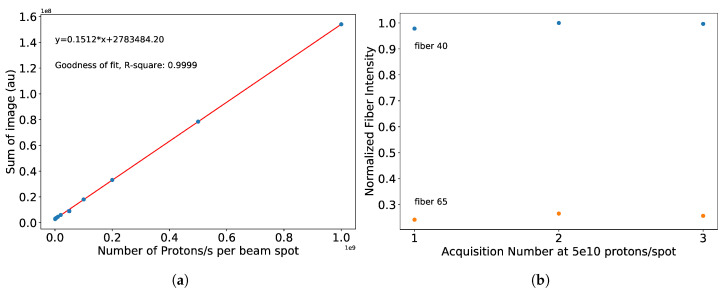
(**a**) Linearity response of the fiber array setup. (**b**) Normalized fiber intensity from each image acquired at the same beam intensity for fibers 40 (highest output) and 65 (lowest output). The intensities are within a few % of each other, showing the reliability of the setup.

In order to measure the linearity of the fiber array, we varied the number of protons delivered per spot and took three consecutive images with the CMOS camera for an exposure time of 10 s. The sum of the entire image of the bundle shows linear behavior from 5 ×$10^{5}$ to 1 × $10^{9}$ protons per spot, as shown in Figure 9. As part of the patient safety measures, the Gantry is limited from delivering beam at lower or higher protons per spot. The dose-dependence information is also available for each fiber and can be extracted using the calibration matrix. For the lowest intensity (5 × $10^{5}$ protons/spot) the standard deviation of the fiber output with the lowest transmission is 2.2% and for the fiber with the highest output is 0.6% of the average of the three acquisitions.

### 3.2. Results from Measurements at METAS

Owing to the versatility of the electron accelerator at METAS, we were able to measure the dose rate response of the fiber array detector. Once the ICT was calibrated to give dose readings, data was acquired with the fiber array detector. The electron beam energy was 18 MeV and a field size of 2 × 2 cm${}^{2}$ was used to uniformly illuminate the entire width of the fiber array.

**Figure 10:**
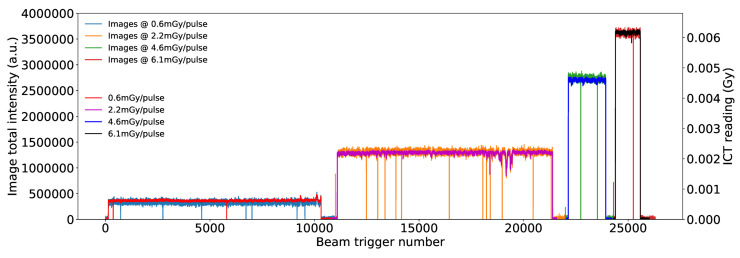
Total intensity of each image taken at different dose rates and the ICT readings in Gy.

Images were acquired for dose rates ranging from 0.6 mGy/pulse to 6 mGy/pulse. An average of 100 background images (i.e., those without beam) were subtracted from each image and the area encapsulating the entire bundle was summed, yielding a single intensity value for a single pulse. Plotted in Figure 10 are the intensity values from each image, for different pulse rates, overlaid with the calibrated ICT readings (V). There are some blank images where the ICT instead reads a current (dose). This discrepancy is due to the occasional delay in the camera trigger to the beam.

**Figure 11:**
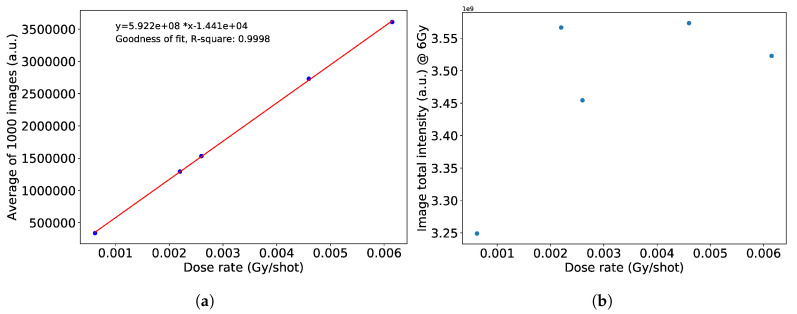
(**a**) The average of 1000 images acquired at each dose rate setting shows a linear behavior. (**b**) The appropriate number of images were summed together to reach a total dose of 6 Gy and plotted with respect to the dose rate. The standard deviation is 6.45%.

We summed the image intensities at each dose rate value, until the corresponding ICT readings reached a total of 6 Gy. Data belonging to beam pulses with blank images were left out of the summations. Plotted in Figure 11 is the detector response at a fixed total dose to varying dose rates per pulse. Although there seems to be a large discrepancy, they are within 6.45% of each other.

**Figure 12:**
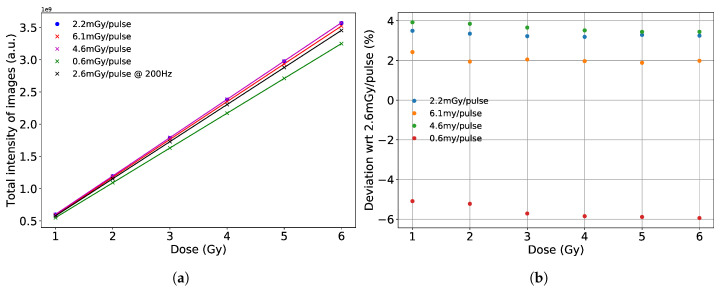
(**a**) Dose response of the detector for different dose rates. (**b**) The deviation with respect to 2.6 mGy/pulse at various dose values.

We also summed the images up to 1–6 Gy, for each dose rate. As mentioned before, data belonging to beam pulses with blank images were left out of the summations. Figure 12 shows the dose response of the detector for different dose rates. The slopes of these responses give a calibration of the fiber array detector setup, with the pco.edge camera, for a variety of dose rates.

## 4. Discussion

The proton study, done at the CPT, characterizes the scintillator-based 1D fiber array detector. Although the fibers have different coupling efficiencies, the position calibration procedure normalizes these differences. The fiber to pixels-on-camera can be matched easily with the position calibration procedure. At the CPT, the fibers in the array show linearity throughout the clinical dose range. Successive images taken at each beam setting have proven that the system response is reliable to a few percent.

The Bragg profiles of each fiber are readily obtainable. The average energy of protons can be extracted from all fibers simultaneously. As described in the introduction, the Bragg peak suppression was expected and is comparable to other results in the literature. The measurements done so far are relative dosimetry.

Next, the detector was taken to the Swiss Federal Institute of Metrology to calibrate, with the sCMOS camera. We were also able to study the dose rate response at a pulsed facility. For the range from 0.6 mGy/pulse to 6 mGy/pulse, the fiber array detector shows a stable performance within 6.45%, and 2.1% for dose rates > 1 mGy/pulse. The detector displays a linear behavior with increasing dose rate. This indicates that the deviation at 0.6 mGy/pulse is not due to the detector setup but due to the calibration of the beam delivery at a low dose rate. Hence, the difference in the calibration slopes. The slope deviations with respect to 2.6 mGy/pulse are within 4% for dose rates > 1 mGy/pulse. Thanks to the accelerator’s ability to deliver pulses at high repetition rates, and to the camera’s frame rate, we were easily able to acquire images at 200 Hz. The performance of the fiber array detector in terms of dose accuracy is comparable to other detectors used in the field such as Gafchromic films [28,29] and Thermal Luminescence Detectors (TLD) [30].

Another functionality is the ability to see the motion of the beam. Triggering the camera to the beam would remove the interplay effect and improve the measurement of the beam motion measured at the CPT. Hence, such a fiber array could also be used in machine Quality Assessment for beam phase-space measurements, where the spot position and shape are measured for several energies. If required, a new camera with higher frame rate capabilities could always be easily integrated. Such cameras already exist commercially.

Stacking these fiber arrays would allow 2D position tracking of protons. Hence, this detector could also be used for pre-treatment quality assurance, where one would need at least 800 × 2 fibers and correction for quenching. The current setup can easily be engineered to be compact, lightweight and scaled up to a higher number of fibers without increasing the cost significantly since a single camera can be used to image several bundles. Stacking several arrays with orthogonal orientation would allow for reconstructing the three-dimensional dose distribution.
